# Utilization of Nano-TiO_2_ as an Influential Additive for Complementing Separation Performance of a Hybrid PVDF-PVP Hollow Fiber: Boron Removal from Leachate

**DOI:** 10.3390/polym12112511

**Published:** 2020-10-28

**Authors:** Hasfalina Che Man, Mohammed Umar Abba, Mohammed Abdulsalam, Raba’ah Syahidah Azis, Aida Isma Idris, Muhammad Hazwan Hamzah

**Affiliations:** 1 Department of Biological and Agricultural Engineering, Faculty of Engineering, Universiti Putra Malaysia, Serdang 43400, Malaysia; gs51611@student.upm.edu.my (M.U.A.); gs50431@student.upm.edu.my (M.A.); hazwanhamzah@upm.edu.my (M.H.H.); 2 Smart Farming Technology Research Centre, Level 6, Blok Menara, Faculty of Engineering, Universiti Putra Malaysia, Serdang 43400, Malaysia; 3 Department of Agricultural and Bioenvironmental Engineering, Federal Polytechnic Mubi, Mubi 650221, Nigeria; 4 Department of Agricultural and Bio-Resources, Ahmadu Bello University, Zaria 810107, Nigeria; 5 Department of Physics, Faculty of Science, Universiti Putra Malaysia, Serdang 43400, Malaysia; rabaah@upm.edu.my; 6 Materials Synthesis and Characterization Laboratory (MSCL), Institute of Advanced Technology (ITMA), Universiti Putra Malaysia, Serdang 43400, Selangor, Malaysia; 7 Department of Chemical Engineering, Faculty of Engineering, Segi Universiti Malaysia, Petaling Jaya, Selangor 47810, Malaysia; aidaisma@segi.edu.my

**Keywords:** nano-TiO_2_, hydrophilicity, flux, antifouling, hollow fiber, boron removal

## Abstract

The continuous increase in anthropogenic activities resulting in an increase in boron concentration in the environment is becoming a serious threat to public health and the ecosystem. In this regard, a hybrid polyvinylidene fluoride (PVDF)-polyvinyl pyrrolidone (PVP) hollow fiber was synthesized with hydrophilic nano-titanium oxide (TiO_2_) at varied loadings of 0, 0.5, 1.0, 1.5, and 2.0 wt% using the phase inversion technique. The resultant membranes were characterized in terms of Scanning Electron Microscopy (SEM), Energy Dispersive X-ray Spectroscopy (EDX), contact angle, porosity, and zeta potential. The permeability flux was assessed using both pure water and leachate; also, rejection performance was evaluated based on boron removal from the leachate. The results revealed that the membrane with 1.0 wt% loading had the highest flux alongside an upturn in boron rejection percentage of 223 L/m^2^·h and 94.39%, respectively. In addition, the lowest contact angle of 50.01° was recorded with 1.0 wt% TiO_2_ loading, and this implies that it is the most hydrophilic. Throughout the experiment cycles, the fiber with 1.0 wt% TiO_2_ loading demonstrated a high flux recovery varying between 92.82% and 76.26% after 9 h filtration time. The physicochemical analysis of the permeate revealed that the boron concentration was significantly reduced to 0.43 mg/L, which is far lower than the discharge limit of 1.0 mg/L.

## 1. Introduction

Accumulated landfill waste generates high-strength leachate, which eventually flows or percolates into the water bodies [1]. The percolated high-strength leachate contains several organic contaminants, including chemical oxygen demand, biological oxygen demand, ammonia, heavy metals, and boron [2,3,4]. Boron is a trivalent metalloid that often exists in the environment either as boric acid or borate ion [5]. It is usually released via anthropogenic activities, as well as through natural weathering processes. The United States Geological Survey (USGS) reported that about 4750 metric tons of boron were generated globally in 2006, which then increased to 9400 metric tons in 2016 due to industrialization and exponential population growth [6]. Some of the adverse effects of boron can lead to problems with the cardiovascular, coronary, nervous, and reproductive systems [7,8]. Boron exhibits a noticeable effect on plants, such as meristematic growth in tissues, disruption of roots and leaves, thickening of leaves, cracking of bark growth, and interference with cell formations, alongside delays in enzyme reactions [9]. Displays of yellowish spots on leaves and fruits along with rapid deterioration and untimely expiration of plants are all due to excess boron [10]. Owing to the potential adverse effects on human health, the Department of the Environment (DOE) of Malaysia and the World Health Organization (WHO) both set regulatory discharge limits to a maximum of 1.0 and 0.5 mg/L in drinking water, respectively [11]. This implies that the need for adequate treatments of industrial effluent to relegate boron concentration to an acceptable discharge limit is unavoidable.

Several methods have been attempted by researchers to eliminate the boron from a generated effluent before discharge. The various methods employed include chemical precipitation [12], activated carbon [13,14], electrocoagulation [15,16], chemical coagulation [17], and reverse osmosis [18] and adsorption [19]. Collectively, some of the most noticeable drawbacks include low adsorption capacity, sludge generation, high cost of chemicals, and frequent fouling during their application. Consequently, this could significantly increase the running cost and operation downtime; as such, it may be unsustainable for large-scale applications. Thus, this necessitated the need for a more robust, cost-effective, and efficient method for boron removal. The membrane-based purification process gained significant attention in the last two decades because of its promising performance in separating boron from wastewater as well as drinking water [20,21]. However, its application is limited by fouling, which usually results in a reduction in flux, excessive energy input, and unsustainable running cost.

Several studies have shown that the fouling effect observed with most of the polymeric membranes is not only due to the foulants’ properties and the operating condition, but intrinsic hydrophobic properties could also be a major factor. Therefore, in order to minimize the fouling challenges, the use of hydrophilic nanoparticle additives to modify the membrane is widely practiced by researchers [22,23,24,25]. Basically, nanoparticles are characterized with a micropore structure and distinctive large surface-to-volume ratio, as well as surface functional groups such as OH [26]. These qualities make it compatible with establishment of a stable matrix structure or a link with most of the polymers commonly used for membrane fabrication [27]. Various nanoparticles such as ZnO [21,22], TiO_2_ [28], Ag_2_O_3_ [24,25], Al_2_O_3_ [29], graphene oxide [30,31], MgO [32], and CuO [33] are commonly applied for modifying polymeric membranes to improve its hydrophilicity properties.

Among hydrophilic additives, TiO_2_ has received an upturn in wider applications, and this might be due to its excellent compatibility, stable linkage with the polymer/co-polymer, and its ability to generate a hydroxyl functional group via the deprotonation process [34,35]. This could support the mitigation of the fouling tendencies of the polymeric membrane [36]. The incorporation of TiO_2_ nanoparticles (NPs) into the polyvinylidene fluoride (PVDF) membrane polymeric matrix makes the surface slightly more negative [37]. Due to their sensing properties, TiO_2_-based structures are also used for the creation of gas-sensing devices [38,39]. Moreover, the blending of TiO_2_ NPs into the membrane matrix was expected to provide an additional advantage in declining fouling propensity [27], thereby making the membrane matrix hydrophilic. For instance, Damodar et al. [36] blended TiO_2_ nanoparticles in a PVDF dope solution, and the results showed that TiO_2_ addition significantly improves the pore size and hydrophilicity of the modified membrane. Oh et al. [40] also noticed a significant improvement in fouling resistance of the PVDF–Ultrafiltration membrane when TiO_2_ nanoparticles were blended into the matrix structure. Likewise, Guangyong et al. [41] and Zhang et al. [42] blended TiO_2_ nanoparticles to improve the antifouling properties of the PVDF matrix.

Despite the potential of the membrane filtration technique for the separation process and the potential of TiO_2_ to improve its hydrophilic properties, information on the application of modified hybrid nano PVDF-polyvinyl pyrrolidone (PVP) for boron separation from leachate remains very scarce. In this regard, this study aims to develop a hybrid PVDF membrane accreted with different levels of TiO_2_ nanoparticles (0, 0.5, 1.0, 1.5, and 2.0 wt%) using the phase inversion (PI) technique. The resultant modified hollow fibers were characterized using Scanning Electron Microscopy (SEM), Energy Dispersive X-ray Spectroscopy (EDX), and goniometric analysis to measure the contact angle. The permeability flux and the performance for boron removal from leachate were evaluated.

## 2. Experimental Methods

### 2.1. Materials

The main membrane-forming material used in this study includes granular PVDF polymer procured from Arkema (Kynar^®^ 760 Inc. Philadelphia, PA, USA); N, N-dimethylacetamide (dimethylacetamide (DMAc); Wako Pure Chemical Industries Ltd., Osaka, Japan) was used without further purification to liquefy the polymer. PVP additive was used as co-polymer to facilitate pore formation, and it was ordered from Sigma-Aldrich (Milwaukee, WI 53,209 USA) (MW = 10,000 Da). TiO_2_ (Degussa P25, average particle size ~21 nm; heat shock pH 7, ≥ 98% analytical grade) was supplied by Sigma-Aldrich (Milwaukee, WI 53,209 United States) and utilized without prior purification. The landfill leachate was collected into an airtight container from a wastewater treatment plant located in Selangor, Malaysia. The collected leachate samples were preserved at 4 °C in a chiller to avert any possible biochemical reactions.

#### 2.1.1. Dope Formulation

Table 1 shows the compositional solutions of PVDF/DMA_C_/PVP/TiO_2,_ prepared under a steady stirring at 250 rpm and 60 °C for a period of 24 h to ensure a homogenous mixture and to eliminate air bubbles. In addition, PVDF granules were added to the prepared solutions and subjected to a higher stirring velocity of 400 rpm at 100 °C (Monotaro; C-MAG HS7, Amagasaki, Hyogo, 660-0876, Japan) for another 24 h. The higher stirring speed and elevated temperature facilitate the dissolving of the granules to give a homogenous dope. Afterward, the final dope solutions were sonicated for 30 min in a water bath at a temperature of 45 °C. The sonicated dope solutions were allowed to cool at room temperature and spun into hollow fibers using the phase inversion technique.

#### 2.1.2. Spinning of Nano-Hybrid PVDF/PVP/DMA_C_/TiO_2_- Hollow Fiber Membrane

The dry-jet wet spinning method was employed to spin the dope fed into the annular spinneret [42]. The extrusion needle of the annular spinneret had an inner and outer diameter of 0.55 and 1.15 mm, respectively. Distilled and tap water were used as the internal and external coagulant, respectively. The pick speed control, collecting drum speed, extrusion rate, air distance, external coagulant temperature, room temperature, and room humidity parameters were all maintained constant at 7 rpm, 9 rpm, 5 mL/min, 10 cm, 25 °C, 29.5 ± 1 °C, and 72.7%, respectively. Then, the spun fibers were soaked in a continuous flow water bath for 24 h to dislodge all the residual solvents. Afterward, post-treatments were administered to the fibers to mitigate shrinkage by dipping fibers in an ethanol solution for 12 h; they were then transferred into a 10 w% glycerol aqueous for another 5 h. To ensure complete dehydration, the treated membranes were air-dried for 24 h at 70 °C. Table 2 presents the spinning parameters of the dry-jet spinning.

### 2.2. Membrane Characterization

#### 2.2.1. Morphological Study

The digital microstructure of the surface and the cross-section of the spun membranes were captured using Scanning Electron Microscopy (SEM) (Model: TM 3000, Hitachi, Tokyo, Japan) at a voltage of 20 kV. Initially, samples for the cross-sectional imaging were frozen in liquid nitrogen. This is to aid sharp fracturing and to ensure a clearer structure. The fractured samples were coated with a thin gold layer and then mounted on the sample’s holder with a carbon tap. Thereafter, the microstructures were examined using an SEM (S-3400, Hitachi, Tokyo, Japan) at an accelerated voltage of 20 kV.

#### 2.2.2. Energy Dispersive X-ray Spectroscopy (EDX) Analysis

EDX analysis of the membrane’s samples with varied loading of TiO_2_ was conducted using a Thermo Scientific instrument; 1 g of the membrane samples at a different dosage of TiO_2_ was used for the EDX analysis using an SEM (TM 3000, Hitachi, Tokyo, Japan) to examine the distribution, dimensions, and elemental composition of nano-hybrid membranes.

#### 2.2.3. Hydrophilic Analysis

The hydrophilicity of the spun fibers was determined based on the water drop surface contact angle using a goniometer (OCA 15EC, Data Physics, Succasunna, NJ 07,876 USA). Initially, a dried membrane sample was attached tightly on the glass panel by using double-sided carbon tape. About 1 µL of deionized water (contact liquid) was dropped on the surface of the membrane by using a microneedle of the goniometer. The instrument automatically captured the angle of the water droplets. For each of the samples, the contact angle was measured in a replicate of 10, and the average value was considered. This procedure is to minimize the level of bias in the data.

#### 2.2.4. Porosity Analysis

The gravimetric method was employed in determining the porosity of the membrane [28]. About 40 cm length of the membrane’s samples, comprising of ten (10) pieces each, were prepared. The opened ends of the samples were sealed using epoxy resin and then soaked in distilled water for 5 h at room temperature (26 ± 1 °C). The soaked fibers were gently removed, and then the traces of water drops on the surface were mopped using dry tissue paper. The soaked–mopped fibers were weighed as wet membrane (M_w_). The soaked membranes were then oven-dried for 24 h at 70 °C, and weight was measured as dry membrane (M_d_). Subsequently, the porosity (ε) of each membrane was determined using Equation (1) [43,44,45].

(1) $$\varepsilon \left(\%\right)=\frac{1}{\rho_{w}}\left(\frac{M_{w}-M_{d}}{V}\right)\times 100$$

where ε is the membrane porosity (%), ρ_w_ is the density of water, M_w_ is the weight of wet membrane, M_d_ is the weight of dry membrane, and V is the volume of the membrane sample.

### 2.3. Evaluation of Membrane Performances

#### 2.3.1. Permeability and Flux Performance

The dead-end permeation device fitted with a membrane module cell was used to assess filtration or permeability efficiency. A suction pressure was provided to the membrane by a peristaltic pump (PLP 6000 manufactured by Dülabo Laborgeräte, Wertheim, Germany). Each of the modules consisted of 25 membrane units with an equal length of 40 cm. The membrane was initially compacted for 30 min at a pressure of 0.4 MPa to ensure a steady permeability, while the successive filtration of leachate was conducted at a lower pressure of 0.3 MPa. The volume of the permeate collected was measured at an interval of 50 min for a total filtration period of 200 min. The flux of the spun membranes filtrating the pure water (J_w_) and leachate (J_L_) was calculated using Equation (2).

(2) $$J=\frac{V}{A\cdot t}$$

where J is the flux in (L/m^2^·h), J_L_, V is the volume of the permeate (L), A is the effective membrane area (m^2^), and t is the time in (h).

#### 2.3.2. Rejection Performance (Re%)

The rejection performance of the spun fibers was evaluated based on boron removal from leachate. The initial concentration of boron (C_p_) in the leachate was 8.2 mg/L, which was determined using an ultraviolet spectrophotometric technique (DR4000U, HACH, Loveland, CO, USA) at an absorbance wavelength of 605 nm. The concentration of the boron in permeate after filtration was also measured as (C_f_). Thus, boron rejection efficiencies were measured using Equation (3).

(3) $$R=\;\left(1-\frac{C_{p}}{C_{f}}\right)\;\times \;100$$

where R is the boron rejection (%), and C_p_ and C_f_ are the boron concentrations in the permeate (mg/L) and feed (mg/L), respectively.

#### 2.3.3. Antifouling and Reusability Analysis

The resultant membranes were subjected to three cycles of filtration with a total operating time of 9 h. Every filtration cycle lasted for 200 min, and was then reapplied for another cycle of filtration after a simple backwash using running tap water for 30 min only. Throughout the experiments, the flux (J_L_) was determined at an interval of 50 min using the corresponding volume of the permeate collected. The antifouling performance of the membranes was evaluated based on the relative flux recovery (%RFR) and flux recovery ratio (%FRR), as expressed in Equations (4) and (5), respectively [41,42,46,47].

(4) $$\mathrm{Relative}\;\mathrm{flux}\;\mathrm{reduction}\;\%\mathrm{RFR}=\;\left[1-\frac{J_{L}}{J_{w}}\right]\times 100$$

(5) $$\mathrm{Flux}\;\mathrm{recovery}\;\mathrm{ratio}\;\%\mathrm{FRR}=\;\left(\frac{J_{w2}}{J_{w}}\right)\times 100$$

where J_L_ is the leachate flux, J_w_ is the pure water flux, and J_w2_ is the remeasured pure water flux after washing, all in (L/m^2^·h).

### 2.4. Zeta Potential Measurement

Using an adjustable gap cell, the zeta potentials of PVDF-PVP and modified PVDF-PVP-TiO_2_ membranes were measured using an electrokinetic analyzer from Anton Paar SurPASS (Berlin Germany). The measurements were carried out using an electrolyte solution purged with nitrogen at 0.1 M KCl. During measurements, 0.1 M NaOH and 0.05 M HCl were used to adjust the pH level.

### 2.5. Analytical Method

The Carmine process was employed to measure the boron concentration present in the leachate before and after treatment. Basically, the boron ver 3 reagents were introduced into a 75 mL concentrated H_2_SO_4_ solution to determine boron concentration. A 2 mL leachate sample and deionized water were correctly pipetted into a 125 mL flask each after 5 min of reaction. A 35 mL boron version 3/H_2_SO_4_ solution was applied to each flask. The prepared sample was placed into the cell holder of the ultraviolet–vis spectrophotometer (DR/4000u HACH, Colorado, USA) at a wavelength of 620 nm to determine the concentration of the leachate sample. All deionized water utilized in the experiments was obtained from the Milli-Q water purification system (18 MQ cm).

## 3. Results and Discussion

### 3.1. Influence of TiO_2_ on Membrane Characteristics

#### 3.1.1. Structural Morphology

**Figure 1:**
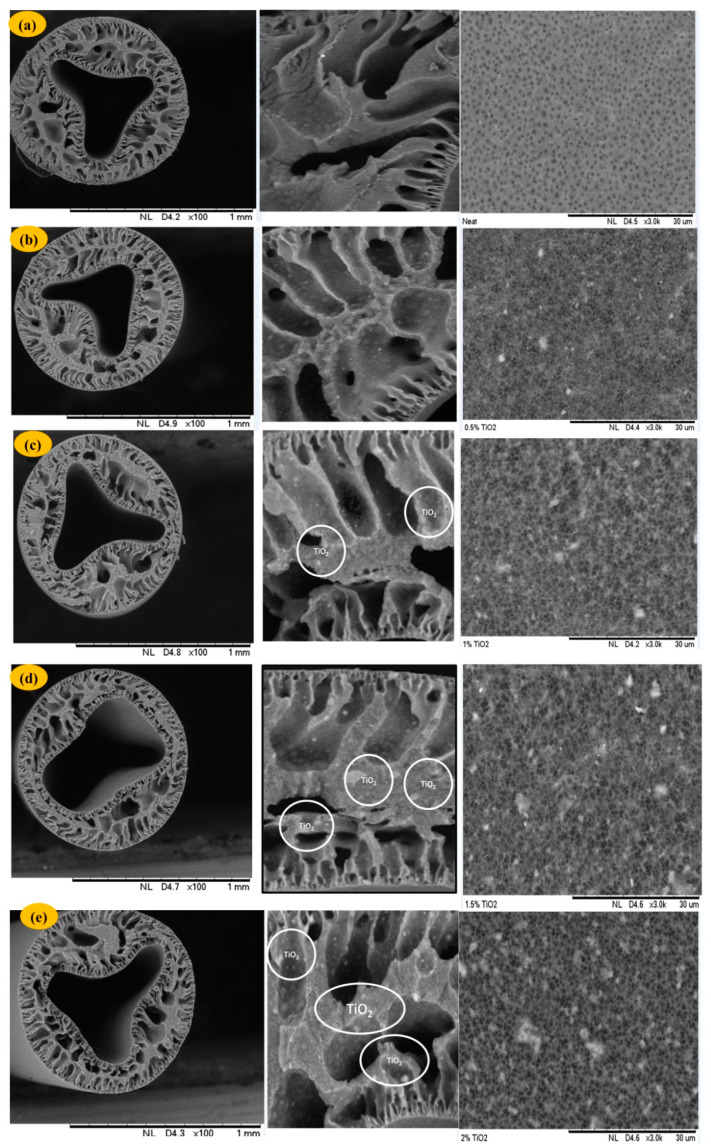
Scanning Electron Microscopy (SEM) pictures (cross and exterior surface) of polyvinylidene fluoride (PVDF) membranes made from separate levels of TiO_2_: (**a**) 0, (**b**) 0.5, (**c**) 1.0, (**d**) 1.5, and (**e**) 2.0 wt% in the presence of polyvinyl pyrrolidone (PVP).

Figure 1 shows the SEM microstructure of the cross-section of the various membranes prepared with a varied TiO_2_ dosage. Blending TiO_2_ nanoparticles into membrane dope results in more pores of greater size on the surface of the membrane [48]. Essentially, as TiO_2_ loading increases (0, 0.5, 1.0, 1.5, and 2 wt%), the finger-like pores of the modified membrane become larger and the number of micropores structures increases considerably [32]; this might be due to the nucleation process alongside crosslinks developed between the TiO_2_ and the polymeric materials [32]. However, homogeneous dispersion of TiO_2_ on the membrane matrix was noted from 0.5 to 1.0 wt%. Conversely, at a greater TiO_2_ concentration (i.e., 1.5 and 2 wt%), these nanoparticles appeared to agglomerate and formed larger nanoparticles, leading to pore blockage and reduction in water flow [49].

#### 3.1.2. EDX Elemental Analysis

**Figure 2:**
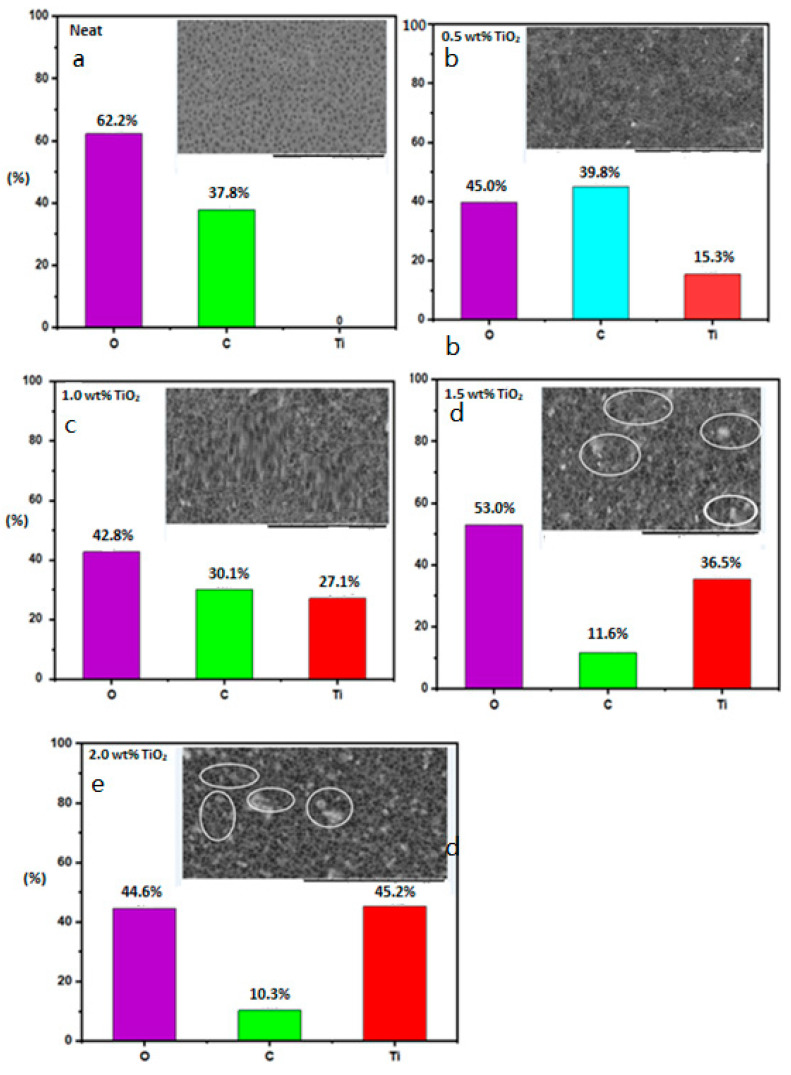
SEM/elemental composition of the PVDF/PVP membrane at different dosages of TiO_2_: (**a**) neat, (**b**) 0.5, (**c**) 1.0, (**d**) 1.5, and (**e**) 2.0 wt%.

Figure 2 shows the SEM and elemental composition of membranes made from the different dosages of TiO_2_—0, 0.5, 1.0, 1.5, and 2.0 wt%, respectively—in the presence of the PVP additive. The neat membrane has only oxygen and carbon in the elemental composition. Membrane accreted with 0.5 wt% TiO_2_ dosage displayed elemental composition of 45% C, 39.8% O, and 15.3% Ti. It was observed that the Ti percentage in the elemental composition increased with an increase in TiO_2_ dosage in the dope. Ti composition increases from 15.3% to 35.5%. However, at higher loading of TiO_2_ (2.0 wt%), the O compositions were reduced to 44.6%. As depicted in Figure 2, 0.5 and 1.0 wt% TiO_2_ loading present a free agglomeration. In contrast, membranes with 1.5 and 2.0 wt% TiO_2_ loading display heterogeneous dispersion and clustering of particles, which led to agglomeration and an increase in viscosity in the dope [50].

#### 3.1.3. Hydrophilicity Analysis

**Figure 3:**
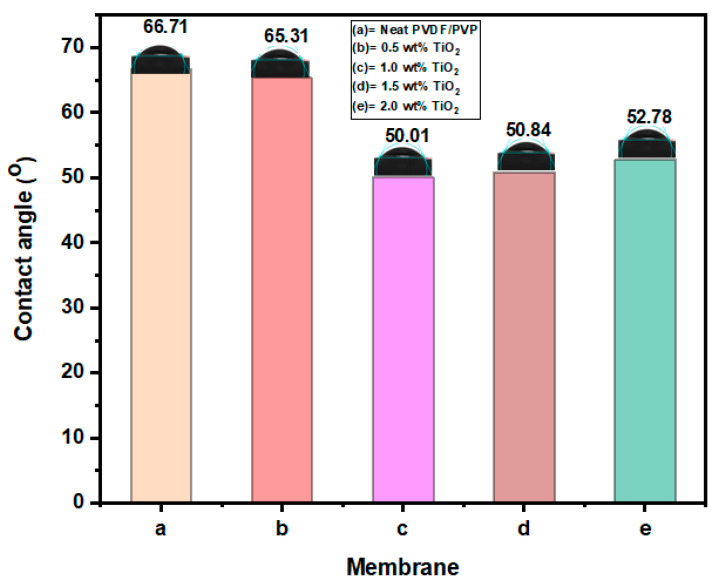
The contact angles of neat PVDF-PVP (**a**) and membranes modified with TiO_2_ (**b**), (**c**), (**d**) and (**e**).

The contact angle is often used to describe the surface hydrophilicity [51]. The contact angle of the neat and modified membrane is shown in Figure 3. The addition of TiO_2_ nanoparticles into the polymeric dope solution produced a clear distinction in both hydrophilicity and flux [52]. The modified membrane with 1.0 wt% TiO_2_ concentration exhibited higher hydrophilicity with the lowest contact angle of 50.01°, while the neat membrane had a contact angle of 66.71°. Further increase in TiO_2_ loading (1.5 and 2.0 wt%) resulted in agglomeration of the nanoparticles within the membrane matrix, a decline in hydrophilicity, and a slight increase in the contact angle. This can be attributed to heterogenous distribution of the nanoparticles, a decrease in surface potential, and blockage of micropores on the membrane surface [53,54]. Based on these results and previous studies, the presence of TiO_2_ nanoparticles in the matrix structure of the modified PVDF membrane improves its hydrophilicity. Furthermore, researchers have also shown that most polymeric membranes with good hydrophilic properties have a higher propensity to resist fouling [48,55].

#### 3.1.4. Membrane Porosity

**Figure 4:**
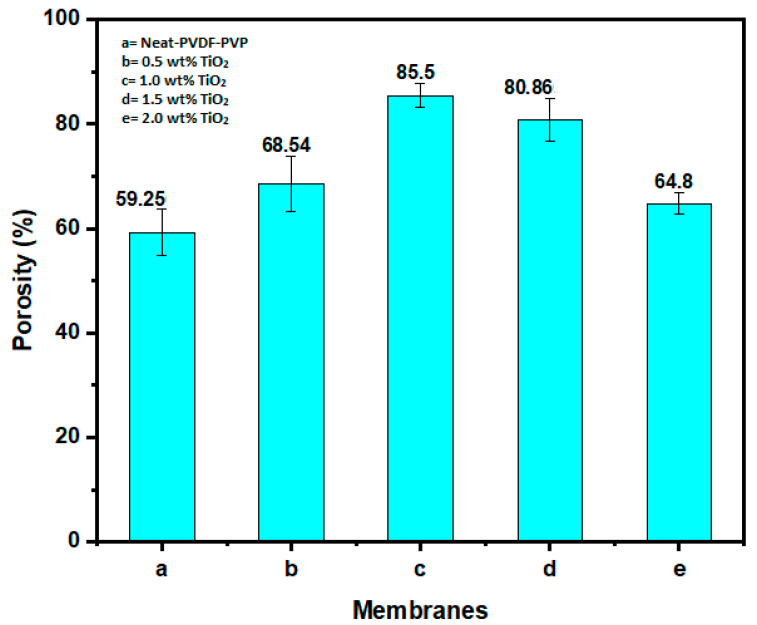
Porosity of neat PVDF-PVP (**a**) and nano-hybrid membranes modified with TiO_2_ (**b**–**e**).

Membrane porosity is greatly influenced by TiO_2_ loading into PVDF dope, as demonstrated in Figure 4. The neat membrane (a) 0 wt% had the least porosity of 59.26%, while the modified membranes had higher values of 68.54%, 85.50%, 80.90%, and 64.90% for the dosages of (b) 0.5, (c) 1.0, (d) 1.5, and (e) 2.0 wt% membranes, respectively. The large jump in porosity from 59.26% to 85.50% at 1.0 wt% loading is due to the incorporation of TiO_2_ nanoparticles in the PVDF matrix, which led to an increase in surface hydrophilicity compared to the neat PVDF membrane [56]. The membrane with 1.0 wt% TiO_2_ loading is more hydrophilic, with a contact angle of 50.01°. The improved hydrophilicity makes the membrane more hydrophilic, which results in a significant increase in porosity and flux [53,57]. However, a further increase in TiO_2_ loading (1.5–2.0 wt%) results in a decline in porosity due to an increase in dope viscosity, which delayed the exchange rate between water and solvent, with resultant lower pore volume in the membrane structure [58,59]. However, the decrease in porosity observed with 2.0 wt% TiO_2_ loading might be due to the effect of agglomeration and high viscosity on the dope [60]. Therefore, it can be presumed that excessive loading of TiO_2_ onto the membrane dope weakens the formation of the pores and could considerably diminish the porosity as well as the permeability of the resultant membrane.

### 3.2. Influence of TiO_2_ on Membrane Performance

#### 3.2.1. Flux

**Figure 5:**
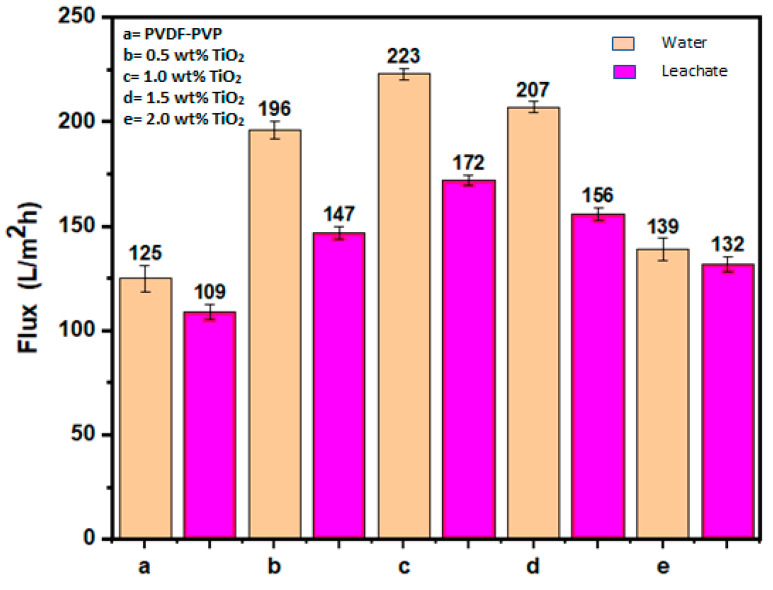
Flux of neat PVDF-PVP (**a**) and nano-hybrid membranes modified with TiO_2_ (**b**–**e**).

The permeate flux of the fabricated membrane at different TiO_2_ loadings is shown in Figure 5. The neat membrane recorded a flux of 125 and 109 L/m^2^·h for pure water and leachate filtration, respectively. In contrast, the modified membrane with the 1.0 wt% TiO_2_ loading recorded more superior flux, with a magnitude of 223 and 172 L/m^2^·h for the pure water and leachate filtration, respectively. This shows that the modification of the membrane matrix using the TiO_2_ nanoparticles significantly improved its hydrophilicity [52]. However, it was noticed that at higher loading of TiO_2_ nanoparticles (1.5 wt%), the flux for the pure water and leachate filtration declined to 207 and 156 L/m^2^·h, respectively. This is due to the agglomeration effect of the nanoparticles on the membrane matrix [32]. This can be attributed to heterogenous distribution of the nanoparticles, a decrease in surface potential, and blockage of micropores on the membrane surface [53,54]. Furthermore, the presence of agglomeration particles within the matrix structure could skew the surface area interaction of the additive (TiO_2_) and cause more roughness on the external part of the membrane [49]. Thus, this could relegate the hydrophilicity and then become more susceptible to fouling even at higher loading [32]. Membrane permeability could also be compromised due to blockage of pores, which hinders the passage of water [49]. Generally, the volume of flux in leachate filtration was noticeably smaller in comparison to pure water flux. This was perhaps due to the presence of more pollutants, particulates, and the colloidal matters in the leachate [22].

#### 3.2.2. Boron Rejection

**Figure 6:**
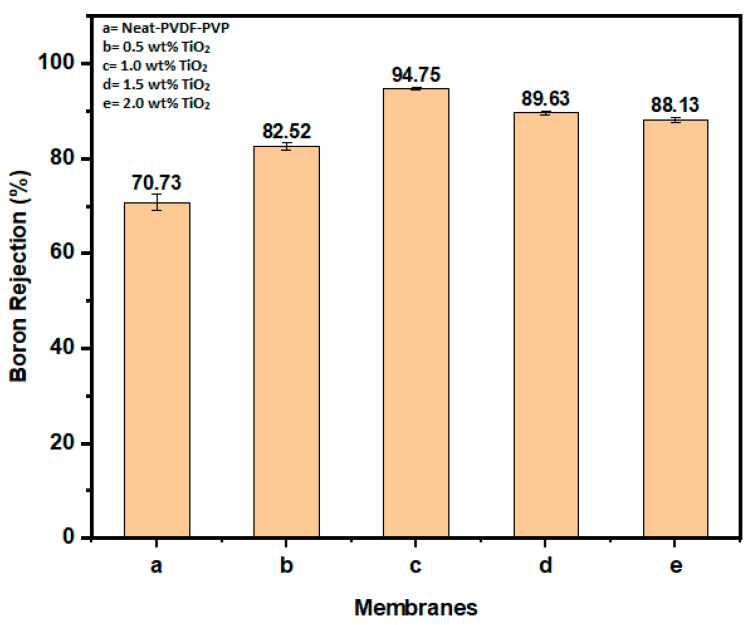
Boron rejection of neat PVDF-PVP (**a**) and membranes modified with TiO_2_ (**b**–**e**).

Boron rejection by neat and modified membranes is shown in Figure 6. The modified membrane with 1.0 wt% TiO_2_ loading demonstrated significant boron removal with 94.75% and 0.43 mg/L concentration, while the neat membrane recorded 72.43% boron removal and 2.4 mg/L concentration. The previous study on the high-performance PVDF-TiO_2_ membrane for water treatment reported a similar finding [61]. Further loading of TiO_2_ nanoparticles in the dope from 1.5 to 2.0 wt% TiO_2_ loading led to a decline in membrane rejection (89% and 88%). This was as a result of changes in the morphology and structure of the membrane surface due to heterogeneous distribution of the nanoparticles [61]. The agglomeration of the particles localized the hydrophilic effect to particular points, while a major part of the matrix was free of the additive (TiO_2_). Therefore, there will be skewed and inconsistent rejection performance of the modified membrane with higher loading. Based on this, it can be deduced that achieving a homogenous mixture of the additive at a certain loading is crucial to achieving good rejection performance.

The addition of TiO_2_ nanoparticles into the PVDF membrane matrix renders the surface more negative, and this might be due to the deprotonation process [36]. Essentially, the deprotonation takes place because the medium of the leachate feed solution is in an alkaline state, which makes the boron in the leachate become ionized into borate ion (B(OH)_4_^−^). In addition, the presence of the TiO_2_ in the matrix facilitates the formation of hydroxyl functional groups (OH) on the surface of the membrane via deprotonation, and this made the membrane surface become more negatively charged [28,32]. The surging ionized borate species towards the membrane surface got rejected due to the like charge repulsion principles [37,62]. Consequently, this process assists in mitigating fouling susceptibility, thereby improving the rejection efficiency.

**Figure 7:**
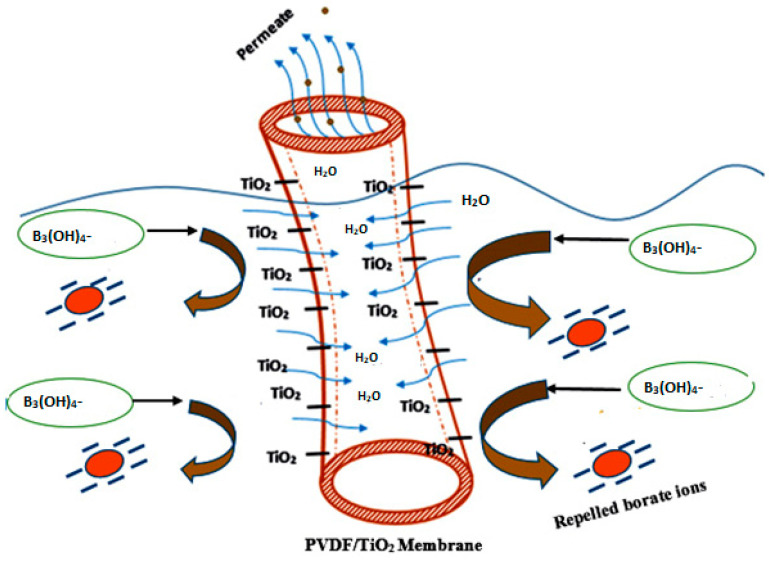
Boron rejection mechanism of the modified PVDF/PVP/TiO_2_ membrane.

Boric acid is hard to be removed via size exclusion due to its low molecular size (0.4 nm), which is smaller than the membrane pore size [63]. The boron in the feed solution (leachate) exists as borate ion (B_3_(OH)_4_^−^) in alkaline conditions. In addition, the presence of dispersed TiO_2_ within the membrane matrix resulted in the formation of negative charges on the membrane surface [37]. Thus, the negatively charged borate ions were repelled upon approaching the membrane surface with like charges, resulting in high boron rejection [62]. The high jump in boron removal is attributed to high negative zeta potential recorded at 1.0 wt% TiO_2_ loading rate, which results in a more considerable amount of hydroxide groups and changes in the overall structure of the membrane. The resultant borate ion repulsion inhibits the accumulation of hydrophobic substances on the membrane surface and pore wall. Based on this rejection phenomenon, separation efficiency and fouling control are enhanced. Figure 7 demonstrates the repulsion mechanism between borate ions (B_3_(OH)_4_**^−^** and the TiO_2_ nano-modified membrane surface while water navigates and passes through membrane pore space and is collected as a permeate.

#### 3.2.3. TiO_2_ Surface Charge

**Figure 8:**
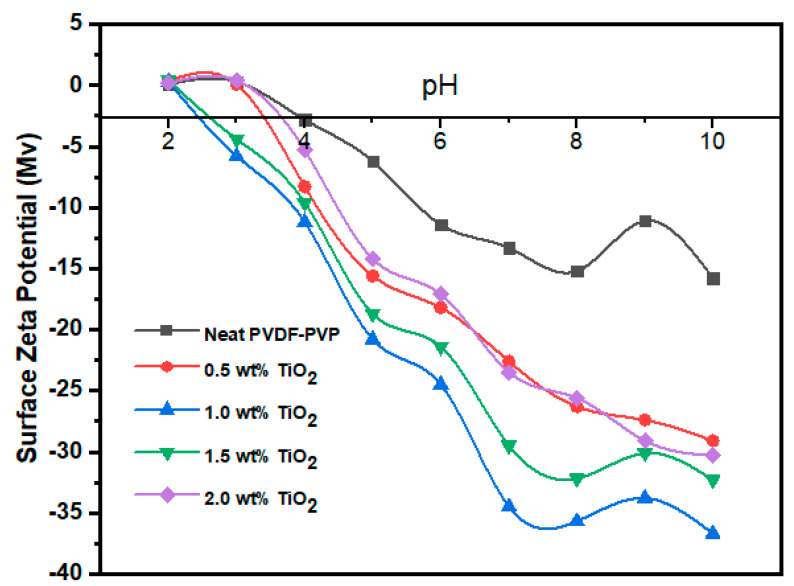
Surface zeta potential of neat PVDF-PVP and modified PVDF/PVP/TiO_2_ membrane.

A zeta potential analyzer was used to assess the surface charge of the PVDF-PVP virgin and modified membranes. Figure 8 presents the surface zeta potential of the fabricated membrane at the varied broad range of 2–10 pH. As depicted in Figure 8, the membrane matrix became more negatively charged alongside increasing TiO_2_ loading. The TiO_2_ exposed to the membrane surface was hydrolyzed to form a hydroxide functional group in the presence of water. The protonation of TiO_2_ resulted in the deprotonation of the membrane surface [64]. Noticeably, the membrane with 1.0 wt% TiO_2_ loading recorded the highest negative zeta potential charge at pH 10, with a value of −36.7 Mv. This is as a result of homogenous dispersion of the nanoparticles within the membrane matrix structure. The high jump in boron removal is attributed to the high negative zeta potential recorded at the 1.0 wt% TiO_2_ loading rate, which results in a more considerable amount of hydroxide groups and changes in the overall structure of the membrane.

Both zeta potential and hydrophilicity are essential parameters in membrane characterization. The membrane surface zeta potential provides information about the possible electrostatic attraction/repulsion, which depends on the quality of the feed stream.

#### 3.2.4. Membrane Fouling Studies

**Figure 9:**
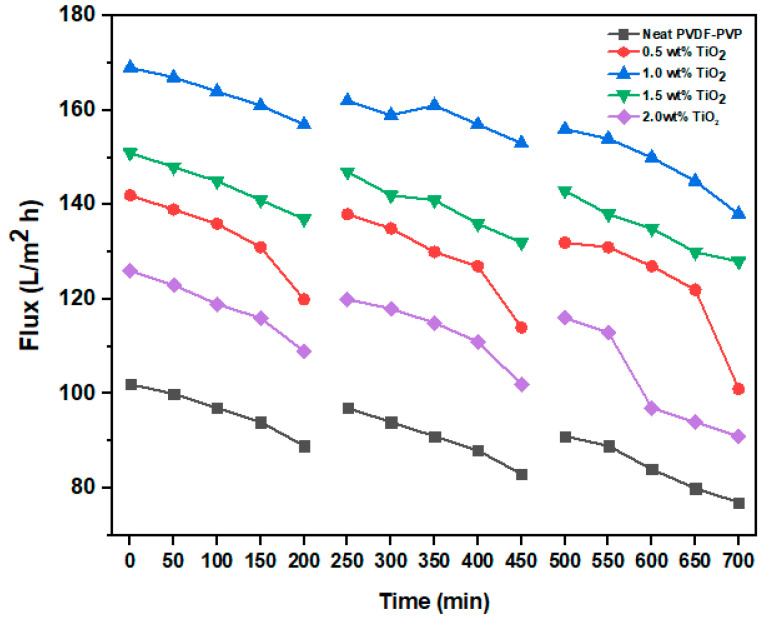
Antifouling and recycling properties of hollow fiber membranes as a function of time.

Figure 9 presents the results of flux in three cycles of filtration with respect to time. Generally, a decline in flux was noticed for both PVDF neat and modified membranes for the dosages of (a) neat, (b) 0.5, (c) 1.0, (d) 1.5, and 2.0 wt% with time due to the deposition of foulants on the membrane surfaces. It was also observed that the neat PVDF membrane had a flux of 89 L/m^2^·h, while the modified membrane (1.0 wt%) had a flux of 157 L/m^2^·h after 200 min filtration. The flux further declines to 77 and 138 L/m^2^·h after the 700 min filtration period, respectively.

**Figure 10:**
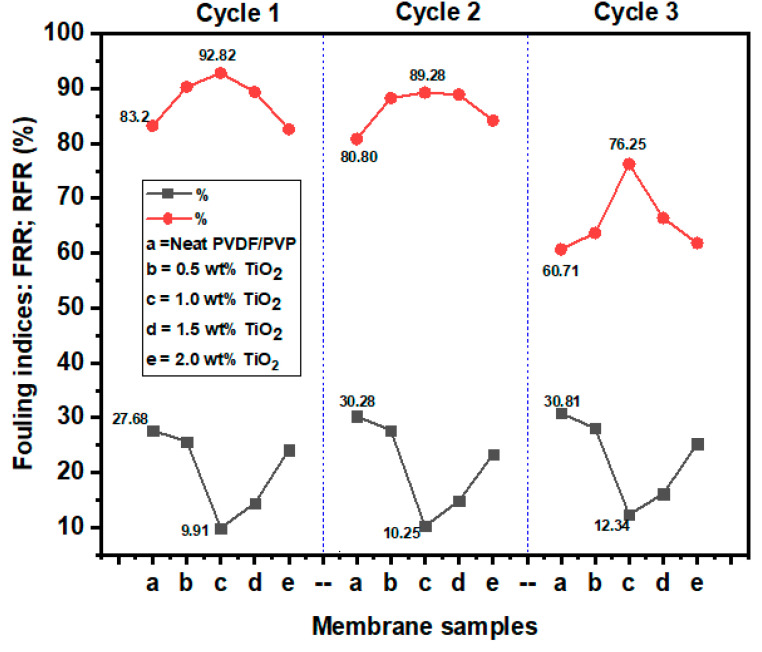
Percent relative flux reduction (%RFR) and percent flux recovery ratio (%FRR) in three filtration cycles using leachate as feed.

Owing to the strong adhesive attraction existing between the interacting interfaces, hydrophobic membranes are more prone to fouling [54]. The incorporation of hydrophilic nanoparticles into the membrane structure can address foulant deposition. On this note, membranes with different dosages of TiO_2_ ((a) 0, (b) 0.5, (c) 1.0, (d) 1.5, and (e) 2.0 wt%) were subjected to three leachate filtration cycles with 200 min for each cycle to investigate the antifouling properties of the membranes. The leachate concentration of the feed was maintained as 8.2 mg/L for each of the cycles. The membranes were physically cleaned under running tap water for 30 min after every filtration cycle. The results of the relative flux reduction (RFR) and flux recovery ratio (FRR) are presented in Figure 10. From the first cycle, the modified membrane (1.0 wt% TiO_2_) had the highest FRR of 92.82%, while the neat membrane had 83.2%. The FRRs of 0.5, 1.0, 1.5, and 2.0 wt% TiO_2_-modified membranes were 90.30%, 89.37%, and 82.58%, respectively. In addition, the fouling resistance performance of the modified membranes also outperformed the neat membrane. The %RFR values for 0.5 wt% TiO_2_, 1.0 wt% TiO_2_, 1.5 wt% TiO_2_, and 2 wt% TiO_2_ were 25.68%, 9.91%, 14.42%, and 24.15%, respectively, compared to 27.68% for the neat membrane. Noticeably, even at the end of the third filtration (cycle 3), the modified membrane with 1.0 wt% TiO_2_ still recorded a high %FRR of 76.25 with %RFR of 12.34.

Generally, the %RFR and %FRR deteriorated along the filtration cycles. The first cycle filtration recorded higher %FRR than the second and third cycles due to fouling. The good antifouling performance of the modified membranes was due to the blending of TiO_2_ nanoparticles into the membrane matrix. This is because hydrophilic nanoparticles have the potential to repel hydrophobic contaminants, thereby enhancing the antifouling property of the membrane [65,66,67].

## 4. Conclusions

The neat PVDF membrane and modified PVDF/TiO_2_ membranes were prepared using the phase inversion method. The nano-TiO_2_ loading was varied from 0 to 2.0 wt%. It was observed from this study that this additive enhances the hydrophilicity, porosity, flux, boron rejection, and antifouling properties of the spun membrane. Importantly, the membrane modified with 1.0 wt% loading demonstrated an unsurpassed performance, with 223 and 172 L/m^2^·h flux for pure water and leachate filtration. In addition, the lowest contact angle and highest boron rejection of 50.01° and 94.75% were obtained with the modified membrane (1.0 wt% TiO_2_), respectively. After three cycles of continuous filtration for 9 h, the modified membrane (1.0 wt%) had the highest flux recovery ratios of 92.82%, 89.28%, and 76.25% respectively. The outstanding performance of the modified membrane (1.0 wt% TiO_2_) was perhaps due to the homogeneous distribution of the nanoparticles on the PVDF-PVP matrix. Therefore, the membrane modified with 1.0 wt% TiO_2_ is strongly recommended for implementation in water and wastewater purification processes aimed at boron removal from landfill leachates.

## Figures and Tables

**Table 1:** Dope chemical composition.

Composition	Polymer (PVDF) (wt%)	Solvent (DMA_C_) (wt%)	Additive PVP (wt%)	TiO_2_ (wt%)
Neat	18.0	77.0	5.0	0.0
Modified (0.5)	18.0	76.5	5.0	0.5
Modified (1.0)	18.0	76.0	5.0	1.0
Modified (1.5)	18.0	75.5	5.0	1.5
Modified (2.0)	18.0	75.0	5.0	2.0

**Table 2:** Spinning parameters.

Parameters	Condition
Spinnerets size	1.15 mm OD/0.55 mm ID
Dope extrusion rate	5 mL/min, 16.67 rpm
Bore fluid composition	water
Air gap	5 cm
Internal/external coagulant	Water
Coagulant bath temperature	Room temperature (25 °C)
Collection drum speed	9 rpm
Washing bath	Water
